# Timing and extent of response in colorectal cancer: critical review of current data and implication for future trials

**DOI:** 10.18632/oncotarget.4747

**Published:** 2015-07-23

**Authors:** Giuseppe Aprile, Caterina Fontanella, Marta Bonotto, Karim Rihawi, Stefania Eufemia Lutrino, Laura Ferrari, Mariaelena Casagrande, Elena Ongaro, Massimiliano Berretta, Antonio Avallone, Gerardo Rosati, Francesco Giuliani, Gianpiero Fasola

**Affiliations:** ^1^ Department of Medical Oncology, University and General Hospital, Udine, Italy; ^2^ Department of Medical Oncology, National Cancer Institute, Aviano, Italy; ^3^ Gastrointestinal Medical Oncology Unit, National Cancer Institute, Napoli, Italy; ^4^ Medical Oncology Unit, San Carlo Hospital, Potenza, Italy; ^5^ Department of Medical Oncology, National Cancer Institute, Bari, Italy

**Keywords:** colorectal cancer, endpoint, response rate, early tumor shrinkage, deepness of response

## Abstract

The identification of new surrogate endpoints for advanced colorectal cancer is becoming crucial and, along with drug development, it represents a research field increasingly studied. Although overall survival (OS) remains the strongest trial endpoint available, it requires larger sample size and longer periods of time for an event to happen. Surrogate endpoints such as progression free survival (PFS) or response rate (RR) may overcome these issues but, as such, they need to be prospectively validated before replacing the real endpoints; moreover, they often bear many other limitations. In this narrative review we initially discuss the role of time-to-event endpoints, objective response and response rate as surrogates of OS in the advanced colorectal cancer setting, discussing also how such measures are influenced by the tumor assessment criteria currently employed. We then report recent data published about early tumor shrinkage and deepness of response, which have recently emerged as novel potential endpoint surrogates, discussing their strengths and weaknesses and providing a critical comment. Despite being very compelling, the role of such novel response measures is yet to be confirmed and their surrogacy with OS still needs to be further investigated within larger and well-designed trials.

## INTRODUCTION

Despite the remarkable survival improvements achieved with modern therapies, unresectable metastatic colorectal cancer (CRC) remains an incurable disease with a 5-year survival rate of approximately 10% [1]. Because of the availability of several active drugs and regimens [2], the assessment of novel treatments may raise new difficulties, including the following:

- if the trial evaluates the effect on survival of a new treatment given after one or more of such active therapies have failed (second-line setting or later), enrolled patients may be less likely to respond to the new therapy; moreover, it may be harder to detect survival benefits in patients with heavily pretreated disease.
- if the trial evaluates a new treatment given upfront in association with standard therapies, (i.e. in first-line setting) its effect on survival:
  - takes several years to be reliably assessed
  - may be diluted by the effects of a long post-progression survival (PPS)
  - may be confounded by the effect of treatments used in second and subsequent lines
  - may be partially counterbalanced by the effect of the same treatment, when used in the control group after progression (crossover).

Furthermore, since the relatively marginal survival benefits that are considered clinically worthwhile by the communities of patients and physicians are narrow [3] and the post-progression survival tends to increase [4], the sample size of randomized trials designed for evaluating new drugs in the metastatic setting is becoming prohibitively large [5].

Overall survival (OS) has been traditionally considered as the most important therapeutic objective for patients with advanced CRC [6]. These issues, however, could be partially overcome if OS was replaced as primary trial endpoint by surrogate endpoints such as progression free survival (PFS) or response rate (RR). These endpoints would allow researchers to assess the beneficial effects of a new treatment in less time, reducing the sample size needed, and avoiding the confounding effects of treatments administered after disease progression.

In this narrative review we discuss the evidence in support and against the use of surrogate endpoints in clinical trials in metastatic CRC. In particular, while commenting on the value of early tumor shrinkage and deepness of response compared with the role of objective response, we discuss if the substitution of the objective response with these novel parameters might replicate its surrogacy.

### Definition of surrogate endpoint

A potential surrogate endpoint is a measure of effect of a specific treatment that should correlate with the real endpoint. It should also reliably anticipate the effect of the treatment on the real endpoint; therefore, it should be biologically associated with both the real endpoint and the treatment. In any case, even if potential surrogate endpoints fulfill all the above criteria, they need to be appropriately (and prospectively) validated before replacing the real endpoints. The validation of a surrogate endpoint remains very challenging. As an example, the role of pathological complete response (pCR) as a surrogate endpoint for prediction of long-term clinical benefit in patients with breast cancer is still largely debated. Even though patients with pCR have an improved survival [7], a recent trial-based meta-regression of randomized studies comparing different neoadjuvant systemic treatments, showed that the therapeutic effect on pCR accounts for only the 9% of the effect on long-term prognosis [8].

As such, the observation that responding patients live longer is not sufficient to establish that an increment in the response rate always translates in an improved survival.

The validation of a surrogate endpoint is both disease- and treatment-specific and requires large randomized controlled trials or meta-analyses of randomized controlled trials. Of note, due to arithmetical reasons, the effects of the treatment on the validated endpoints are always greater than the effects on the real endpoint. Unfortunately, while empirical criteria for surrogate endpoint definition are still debated, clinical decisions based on invalid surrogate endpoints may have public health consequences [9–13].

### Time-to-event endpoints as surrogates of overall survival

Besides OS, other time-to-event endpoints such as PFS and time-to-progression (TTP) may be useful in later stages of drug development, providing valuable information with the advantage of being unaffected by subsequent therapies [6]. In the advanced CRC setting, PFS was validated as a surrogate for OS by strong evidence when using chemotherapy alone [14, 15]. An analysis conducted in 4,352 patients diagnosed with advanced CRC and treated with chemotherapy in 13 trials showed a correlation coefficient (R) between treatment effects on PFS and on OS of 0.99 (95%CI, 0.94 to 1.04) when all trials were considered. Moreover, the same analysis showed that a hazard ratio (HR) of at least 0.77 in terms of PFS would predict a benefit in terms of OS [14]. Based on these results, PFS was chosen as primary endpoint in the majority of first-line randomized trials.

The same surrogacy, however, appeared to be less relevant when combining chemotherapy with Vascular Endothelial Growth Factor (VEGF)-inhibitors or Epidermal Growth Factor Receptor (EGFR)–inhibitors, although the data are still unclear. A meta-analysis of 50 trials with overall 22,736 patients showed a correlation within PFS and OS of 0.52 in trials containing monoclonal antibodies [16]. Notably, the analysis showed a strong correlation in 7 cetuximab- or panitumumab-based trials including 1,335 patients (R 0.96), whereas the correlation within PFS and OS was lower (R 0.45) in 11 bevacizumab-based trials with a global number of 3,310 patients. Such findings were recently confirmed also by Ciani's meta-analysis which showed that surrogacy relationships observed between PFS and TTP vs. OS in selected settings may not apply across other classes or lines of therapy [17].

The use of PFS as primary endpoint however has some limitations, such as the timing of tumor assessment and the difficulties related to the categorization of patients without measurable disease. As a matter of fact, PFS needs accurate monitoring of tumor assessment, and unaware independent radiologists should revise the imaging. Moreover, the time of disease progression detection may depend on the reassessment time schedule. In fact, estimates of PFS are highly dependent on the time in which we look for progression. In a two-arm study, for example, comparison of PFS across treatment arms was often based on assessment intervals that differed across arms [18]. Definition of PFS depends also on the date of randomization. That information is relevant considering that PFS interval is alternately calculated from the start of maintenance therapy in some cases or from the start of first-line treatment in others.

As a consequence of the suboptimal role of PFS, composite or alternative endpoints such as duration of disease control or time to failure of the treatment strategy have been recently proposed [19, 20]. For the time being, however, the value of composite or alternative endpoints is merely conceptual.

### Objective response and response rate as surrogate endpoints of overall survival

As every clinician would intuitively advocate, it is reasonable to expect a better outcome in patients who respond to treatment. Tumor response and clinical benefit have always been considered closely associated in most solid tumors, including advanced CRC [21]. This hypothesis was first confirmed by a meta-analysis encompassing individual patient data from 3,791 metastatic CRC patients treated with fluoropyrimidine-based chemotherapy and enrolled in 25 randomized trials [22]. The meta-analysis showed that tumor response was a valid surrogate of the effect of fluoropyrimidine-based chemotherapy on survival, independently from other clinical factors. However, the analysis also underlined that a treatment lowering the odds of failure to respond by 50% would be expected to decrease the odds of death by only 6%, since the correlation was very low [22]. Considering all available randomized controlled trials across different drug classes and lines of therapy, tumor response should not be regarded as an acceptable surrogate end point for OS [17].

The RR defines the proportion of patients with tumor shrinkage of a predefined value and for a minimum period of time. The RR is usually measured from the time of initial response until documented tumor progression and it directly reflects drug activity. It is typically used as a primary endpoint in phase II trials, in which it is sometimes coupled with the response duration. In randomized phase III trials, RR is usually considered a secondary endpoint.

Noteworthy, the objective response (OR) observed in a patient can be used with 3 different aims:

- as an indication that the tumor was sensitive to the regimen administered;
- as an indication of clinical benefit obtained by the patient from the treatment in terms of improved quality of life and, consequently, in terms of prolonged life time;
- as an indication that the treatment should be continued because the patient is responding to it.

The first aim denotes the activity of the regimen, and its associated statistics, the RR (i.e. the proportion of responses) is commonly used to decide if that regimen warrants further studies in patients with the same type of tumor. The second aim qualifies OR as a potential surrogate endpoint of survival. If its prognostic role is shown to be independent of the treatment that induced the response (Prentice criteria [23]) or a correlation is observed between the effects of the treatment on RR and those on OS across different trials (meta-analytic validation [13, 24]), RR can be safely accepted as a surrogate primary endpoint in clinical trials [25]. The third aim represents an extension of the concept of surrogacy since it implies that further benefits can be obtained by prolonging the treatment.

Of note, the magnitude of clinical benefit not only depends on the rate of response but also on disease extension and anatomic location (e.g. visceral versus not visceral responses [26]). Responding patients with liver or lung metastases may have a greater clinical benefit compared to responders with secondary nodal and bone involvement [27, 28]. Therefore, it comes as no surprise that the correlation between RR and OS tends to be weaker in metastatic breast cancer, which is frequently associated with bone, cutaneous or node metastases, compared with the correlation observed in metastatic colorectal cancer, which preferentially leads to visceral metastasis [28].

Overall, the relationship between OR and survival in solid tumors and the potential role of response as a surrogate of survival has great relevance. Nevertheless, such issues have only been tackled in the last 15 years, during which the methodological and statistical concerns they involve have been addressed.

### Classical assessment of tumor response: WHO and RECIST

Measuring the tumor response to a given treatment is often challenging, although the problems may differ from daily practice to clinical trials. In everyday clinical practice, whether a treatment is deemed active or not is based on several considerations, including subjective judgment and arbitrary evaluations, more focused on patient's profile rather than on his/her metastatic lesions. Conversely, in clinical trials objective and reproducible criteria are required to assess and classify tumor response, and usually the evaluation is based on the radiological assessment of measurable/evaluable tumor lesions.

While in the historical World Health Organization (WHO) criteria a bidimensional measurement of tumor lesions was pursued [29], the Response Evaluation Criteria In Solid Tumors (RECIST) criteria introduced the use of unidimensional measures for overall evaluation of tumor burden [30] (Table 1). In fact, based on the assumption that the simple sum of the maximum diameters of individual tumor is more linearly related to cell death than the sum of the bidimensional products, the analysis of data from 569 cancer patients have demonstrated that the unidimensional measurement was a sufficient tool to assess change in solid tumors [31]. Both the WHO and RECIST criteria guidelines, conceived with the purpose of creating a more objective, reproducible and comparable tumor response assessment, were developed in the era of cytotoxic agents. Subsequent versions of these criteria, an effort to fine-tune the common language used by researchers in a rapidly changing treatment landscape, also had limitations. Although RECIST 1.1 criteria are extensively applied in clinical trials [32], they suffer from the significant inter-variability among readers [33], the difficult evaluation of the margins of ill-defined or irregular lesions, and the lack of objective evaluation of non-measurable disease [34]. Moreover, RECIST criteria do not consider the time dimension.

**Table 1:** Summary of major differences between WHO and RECIST criteria

CHARACTERISTIC	WHO	RECIST 1.0	RECIST 1.1*
**MEASURABILITY OF LESIONS AT BASELINE**	MEASURABLE LESIONS		
	Bidimensional: product of longest diameter and greatest perpendicular diameter	Unidimensional: longest diameter, size with conventional techniques/clinical examination≥20 mm ≠ spiral CT≥10 mm lymph node not mentioned	Size: CT/clinical examination ≥10 mm lymph node - ≥15 mm short axis for target - 10–15 mm short axis for non-target - <10 mm non-pathologic
	NONMEASURABLE/EVALUABLE		
	Accepted (e.g., lymphangitic pulmonary metastases, abdominal masses)	Nonmeasurable: all other lesions, including small lesions. Evaluable is not recommended.	Included on bone lesions and cystic lesions.
**OBJECTIVE RESPONSE**	MEASURABLE DISEASE or TARGET LESIONS		
	MEASURABLE DISEASE: change in sum of products of longest diameters and greatest perpendicular diameters. no maximum number of lesions specified	TARGET LESIONS change is sum of longest diameters maximum of 5 per organ up to 10 total (more than one organ)	5 lesions (2 per organ)
	CR: disappearance of all known disease, confirmed at ≥4 weeks	CR: disappearance of all target lesions, confirmed at ≥4 weeks lymph node not mentioned	CR lymph nodes must be
	PR: >50% decrease from baseline, confirmed at ≥4 weeks	PR: >30% decrease from baseline, confirmed at ≥4 weeks	
	PD: >25% increase of one or more lesions, or new lesions	PD: ≥20 mm, 20% increase over smallest sum observed, or new lesions	PD: 20% increase over smallest sum on observed and at least 5 mm increase or new lesions
	NC: neither PR nor PD criteria met	SD: neither PR nor PD criteria met	
	NONMEASURABLE DISEASE or NONTARGET LESIONS		
	CR: disappearance of all unknown disease, confirmed at ≥4 weeks	CR: disappearance of all target lesions and normalization of tumor markers confirmed at ≥ 4 weeks	
	PR: estimated decrease of 50% confirmed at ≥4 weeks	-	
	PD: estimated increase of ≥ 20 mm, 25% in existent lesions of appearance of new lesions	PD: unequivocal progression of nontarget lesions or appearance of new lesions	Unequivocal progression should not normally trump target disease status. It must be representative of overall disease status change, not a single lesion increase
	NC: neither PR or PD criteria met	Non-PD/Non-CR: persistence of one or more nontarget lesions and/or tumor markers above normal limits	
**OVERALL RESPONSE**	Best response recorded in measurable disease	Best response recorded in measurable disease from treatment start to disease progression or recurrence	
	NC in nonmeasurable lesions will reduce a CR in measurable lesions to an overall PR	Non-PD/Non-CR in nontarget lesions will reduce a CR in target lesions to an overall PR	
	NC in nonmeasurable lesions will not reduce a PR in measurable lesions	Non-PD/Non-CR in nontarget lesions will not reduce a PR in target lesions	

* Major changes from RECIST 1.0 to RECIST 1.1

WHO = World Health Organization; RECIST = Response Evaluation Criteria in Solid Tumors; CR = complete response; PR = partial response; PD = progressive disease; NC = no change; SD = stable disease.

RECIST criteria cut-offs remain arbitrary, since the 30% threshold in establishing reduction has no solid anatomic or biological background and often requires adaptation when measuring metabolic active lesions. Furthermore, traditional RECIST criteria perform poorly when evaluating the efficacy of cytostatic drugs, because the real benefit is not captured from the variation of tumor burden [35–37]. Additionally, specific target therapies may induce increases in size in responding metastatic lesions because of internal hemorrhage, necrosis or myxoid degeneration [38], causing major interpretative issues. Notable examples of this phenomenon have been documented with the use of bevacizumab [39] or regorafenib [40]. Accordingly, collaborative efforts have already been made to evaluate more accurately the morphological changes of the metastatic lesions [41, 42] and to correlate the radiological changes with pathological responses in those resected [40, 43].

The precise definition of pattern of response has been further challenged with the introduction of immunomodulatory molecules. Ipilimumab, for example, produces distinct patterns of response: regression of baseline lesions with no new lesions, stable disease followed by a slow, steady decline in tumor burden, delayed response after an initial increase in tumor burden and response after the appearance of new lesions. As for the timing of tumor assessment, it is usually required at the end of induction dosing period and at least 4 weeks later (response confirmed) [44]. Ongoing trials testing PD-1/PD-L1 inhibitors in gastrointestinal cancers [45] will most certainly complicate the current landscape [46]. Notably, immune-related response criteria are being used in trials and clinical practice even if not prospectively validated yet.

### Developments in the assessment of tumor response: metabolic and biologic response

To overcome some of the limits of the RECIST criteria, functional [47, 48] or metabolic criteria, such as the Positron Emission Tomography (PET) Response Criteria in Solid Tumors (PERCIST) [49] as well as attenuation measurements reflecting tumor necrosis [43] and measurement of the viable parts alone [50] have been proposed.

Early changes in tumor metabolism have shown to significantly predict long-term outcomes during preoperative treatment of patients with liver metastases from CRC [51]. In 33 patients treated with FOLFIRI plus bevacizumab, between the first PET scan (obtained before the beginning of treatment) and the second PET scan (acquired after 1 cycle of chemotherapy in 31 patients and after 2 cycles in 2) there was a notable decrease of all the patient-based PET measures with a median change of −33.9% (range, −78.3 to +54.0) for the highest SUV max. After a median follow-up of 30 months, patients defined as responders with PET outcome had significantly longer PFS and OS than non-responders. Early response evaluated by PET has shown to predict PFS and OS also in patients with metastatic CRC receiving third-line cetuximab-based therapy [52].

Standardization of methodology for PET-based response evaluation is needed in order to compare results achieved from different studies [53]. Recent studies have suggested that an early assessment with PET/CT scan for example as early as 2 weeks after chemotherapy, was associated with the anatomic response and correlated with survival. Such findings support further research to validate the use of early PET response as a surrogate of long-term outcome in patients with metastatic cancer [54].

Even though both RECIST and PERCIST criteria seem to correlate with survival [55], the morphologic and metabolic response agreement is poor [56].

Exploring similarities and differences between metabolic and RECIST response might be a useful way to implement understanding of tumor biology as well as the treatment efficacy, especially after introduction of new drugs such as bevacizumab and cetuximab. Of note, differences were observed analyzing the correlation between response rate and KRAS mutational status of patients with metastatic CRC treated with irinotecan and cetuximab. As a matter of fact, a large number of patients harboring K-RAS mutation were found to have a metabolic but not a clear morphologic treatment response, probably due to a more sensitivity of PET/CT scan [54–56]. Since computer tomography (CT) scan enhancement is related to the amount of blood perfusing the tumor, it may be speculated that the attenuation in tumor density observed during treatment can be attributed to tumor devascularisation and necrosis. To evaluate the tumor response, the Choi criteria [57] combine changes in tumor size with the attenuation in density expressed in Hounsfield units (HU). According to these new criteria, a decrease ≥10% in the sum of sizes together with a decrease ≥15% in the mean attenuation of target lesions measured by CT with injection of contrast material accounts for a partial response (PR), whereas progressive disease (PD) is defined as *a* ≥ 10% increase in size not fulfilling the PR criteria for density. However, both size and density of liver lesions may be difficult to determine because of devascularisation of the lesions during treatment, especially in patients who underwent locoregional treatment. The European Association for the Study of the Liver (EASL) criteria take into account only the portion of the lesion enhanced after injection during the arterial phase (the viable portion) to assess the efficacy of focal therapies. Recently, the combination of the RECIST with the EASL criteria has led to the development of the modified RECIST criteria, which evaluate only the percentage change in the sum of the diameters of the viable portions [58, 59].

### New response parameters: timing

It is reasonable to hypothesize an inverse relationship between the time a tumor takes to shrink when treated and its sensitivity to that specific treatment. Moreover, it might be suggested that patients who respond quickly to a treatment may experience better outcomes than those with slower response or disease stabilization. Although the evidence supporting such hypothesis is scanty, the possibility that early tumor shrinkage (ETS) becomes a new valuable parameter in clinical trials is attractive.

This hypothesis has been already tested in the breast cancer model. In the GeparTrio phase III study, early tumor response was assessed after two cycles of neoadjuvant treatment with docetaxel, doxorubicin, and cyclophosphamide. Overall, 22.2% of patients who experienced an early response achieved pathological complete response (pCR), compared with only 5.6% of those who have no response after two cycles of chemotherapy [60]. Accordingly, a meta-analysis of 7 neoadjuvant studies showed that response to the first 2 to 4 cycles of preoperative chemotherapy strongly correlated with survival outcomes, especially in patients with high aggressive tumors [61].

The definition of ETS, however, is not consistent among different studies. Published data showed that KRAS wild-type metastatic CRC patients who experienced a relative decrease of tumor size >9.66% after 6 weeks of cetuximab-based treatment had a significantly longer median OS compared with all other patients (74.9 weeks versus 30.6 weeks, *p* = 0.0000025); therefore, ETS might be defined as the decrease in tumor size of at least 10% at the very first radiological assessment since the beginning of treatment [62].

To verify if faster tumor shrinkage may be used as a prognostic factor, a retrospective analysis of 113 irinotecan-refractory patients enrolled in four clinical trials (BOND, EVEREST, SALVAGE and BABEL) not only showed that the decrease in tumor size was greater in KRAS wild-type patients when compared to mutants (mean relative change −13.73% versus +2.27%, *p* < 0.001), but also that the rapid tumor shrinkage correlated with a better outcome [62]. In particular, patients with a tumor size decrease of at least 10% at the 6-week radiological assessment had a median PFS of 36 weeks (95%CI 34.6–37.4) compared with 12 weeks (95%CI 22.9–39.7) in patients who did not exhibit an early tumor response (* p* < 0.001). Similarly, median OS was 65.9 weeks (95%CI 41.3–90.4) in patients who had an early tumor size reduction and 31.3 weeks (95%CI 22.9–39.7) in those who did not. In the Cox regression analysis, ETS was a strong predictor for survival (HR 0.42) [62]. In another retrospective study, radiological data of 329 patients enrolled in the BOND trial were reviewed to verify if ETS of at least 10% correlated with RR and survival parameters. In the univariate analysis an excellent correlation between ETS and RR was reported ( *p* < 0.005). Moreover, patients with ETS had a significantly longer TTP (HR 0.22; 95%CI 0.17–0.32, *p* < 0.001) and longer median OS (HR 0.24; 95%CI 0.20–0.43, *p* < 0.001). In multivariate analysis, ETS was confirmed to be the strongest predictor of TTP (HR 0.22, 95%CI 0.16–0.31, *p* < 0.001) and OS (HR 0.21, 95%CI 0.14–0.32, *p* < 0.001) [63].

Similarly, a retrospective analysis of CRYSTAL and OPUS trials showed a significant association between ETS (in this case defined as an early shrinkage of at least 20%) and PFS ( *p* = 0.027 for CRYSTAL and *p* = 0.004 for OPUS), but not OS (* p* = 0.573 and *p* = 0.546, respectively) in patients exposed to cetuximab [64]. It has been argued that the lack of association of ETS with OS may be due to the crossover effect after disease progression. In this analysis, tumor shrinkage was more pronounced in KRAS wild-type patients receiving chemotherapy plus cetuximab compared to those exposed to chemotherapy alone. Moreover, ETS entailed longer PFS in KRAS wild-type patients treated with cetuximab compared to non-ETS (14.1 months vs. 7.3 months, HR 0.32, *p* < 0.001 in CRYSTAL; 11.9 months vs. 5.7 months, HR 0.22, *p* < 0.001 in OPUS) and longer OS (30 months vs. 18.6 months, HR 0.53 *p* < 0.01 in CRYSTAL; 26 months vs. 15.7 months, HR 0.43, *p* < 0.006 in OPUS). Benefit derived from ETS was certainly smaller for patients treated with irinotecan-based chemotherapy alone and marginal for those treated with FOLFOX-4. In summary, this retrospective analysis showed that early tumor assessments might provide predictive information for long-term outcome of metastatic CRC patients exposed to first-line chemotherapy in combination with cetuximab.

Also, a different tumor volume algorithm was developed to provide a better approximation of the real tumor volume using both the longest and the longest orthogonal diameters of a target lesion. Accordingly, patients treated with cetuximab plus either CAPIRI or CAPOX who experienced at least 20% of tumor shrinkage at 8 weeks had higher RR (82% versus 19%, *p* < 0.001), longer median PFS (8.9 versus 4.7 months, *p* < 0.001) and better OS (31.6 versus 15.8 months, *p* = 0.005) when compared with non-responders [65]. Other confirmatory analyses from trials enrolling metastatic CRC patients treated with cetuximab plus chemotherapy (FIRE-1, CIOX, OPUS, and CRYSTAL trials) have been presented [66]. Moreover, Mansmann and coll. demonstrated that the tumor volume algorithm might be more accurate in predicting individual patients’ PFS and OS than RECIST-based tumor assessments [67].

A large meta-analysis from the ARCAD database including radiological data from 11,987 patients enrolled on 15 randomized first-line phase III trials (with 8 trials evaluating targeted therapies) assessed the correlation between Early Objective Tumor Response (EOTR), measured at 6, 8, or 12 weeks, and OS or PFS [68]. Median OS was significantly longer in patients with EOTR at 6 weeks assessment compared to the others (21.7 months versus 16.5 months, HR 0.64, 95%CI 0.58–0.70, *p* < 0.0001), regardless of the use of targeted therapies (HR 0.68 versus HR 0.61). Accordingly, median PFS was 8.4 months in patients with EOTR at 6 weeks versus 7.0 months in patients without EOTR (HR 0.79, 95%CI 0.73–0.85, *p* < 0.0001). EOTR at 8 and 12 weeks was also associated with longer survival outcomes. The authors suggested that early response measured at 6, 8 or 12 weeks might be considered a strong independent predictor for both OS and PFS.

The prognostic role of ETS when antiangiogenic drugs are used upfront in combination with chemotherapy has also been studied. In the TRIBE trial, 508 metastatic CRC patients were randomized to receive bevacizumab with either FOLFIRI or FOLFOXIRI for up to 12 cycles [69]. Patients in the experimental arm achieved a significantly longer PFS (12.1 months versus 9.7 months; HR 0.77, 95%CI 0.64–0.93, *p* = 0.006) and a significant increase in RR (65% versus 53%, *p* = 0.006). Recently, 441 patients were evaluated for ETS (shrinkage cut-off >20%) that was more frequently observed in patients assigned to the FOLFOXIRI plus bevacizumab arm (62.7% versus 51.9%, *p* = 0.025). Moreover, ETS was associated with prolonged median PFS (17.1 months versus 11.5 months, HR 0.65; 95%C, 0.49–0.86, *p* = 0.030], and longer median OS [31.9 months versus 21.9 months, HR 0.63; 95%CI 0.48–0.83, *p* = 0.001] [70].

A pool-analysis of 21 trials including 16 phase III, 4 randomized phase II, and 1 non-randomized phase II studies showed that patients with ETS had significantly better outcomes in terms of PFS (HR 0.57, 95%CI 0.47–0.68, *p* < 0.001, heterogeneity I^2^ 68%) and OS (HR 0.58, 95%CI 0.53–0.64, *p* < 0.002, heterogeneity I^2^ 41%) compared with those patients with no ETS, regardless of the biologic agent used in first-line treatment. However, no correlation between ETS and OS was found (R 0.37, 95%CI −0.31–0.78, *p* 0.28) and the reliability of ETS as a potential surrogate endpoint was not confirmed [71]. Recently, a secondary analysis of a randomized trial investigating the role of cetuximab combined with FOLFOX in 138 liver-limited KRAS wild-type CRC patients [72] confirmed the impact of ETS achieved at 8 weeks on clinical outcome [73].

In PEAK trial achievement of ≥ 30% versus < 30% ETS at week 8 was associated with longer median PFS (12.8 vs 9.7 months; HR: 0.54 [95% CI: 0.36–0.80]; *p* = 0.0019). Similar results were seen when combined data were analyzed using the ≥20% ETS cut-off. Of note, more patients treated with panitumumab versus bevacizumab had ≥30% ETS at week 8 [74].

Data about the impact of ETS on PFS and OS in patients exposed to chemotherapy plus bevacizumab or cetuximab are summarized in Table 2 [53–56, 61, 75, 76].

**Table 2:** Impact of ETS on PFS and OS in patients treated with chemotherapy +/− cetuximab or bevacizumab

First Author	Trial	Definition of ETS	Regimen	% of pt with ETS	mPFS (mo)according to ETS	HR PFS ( *p*)	mOS (mo)according to ETS	HR OS ( *p*)
De Roock 2008 [62]	BOND SALVAGE EVEREST BABEL	**6wk**^∼^**10%**	CT +/− Cx	NR	9 vs 3	HR NR (*P* < 0.0011)	16.5 vs 8 (19 vs 7.5 in KRAS wt)	HR NR (*P* < 0.0012)
Piessevaux 2009 [63]	BOND (phase III)	**6wk, ≥10%**	Irinotecan +/− Cx	34.3	7 vs 1.6	0.22 (*p* < 0.001)	13.4 vs 7.3	0.24 ( *p* < 0.001)
Piessevaux 2013 [64]	CRYSTAL (phase III)	**8wk, ≥20%**	Folfiri	49	9.7 vs 7.4	0.58 (*p* < 0.001)	24.1 vs 18.6	0.71 (*p* < 0.006)
			Folfiri/Cx	62	14.1 vs 7.3	0.32 (*p* < 0.001)	30 vs 18.6	0.53 (*p* < 0.001)
	OPUS (phase II)		Folfox4	46	7.2 vs 7.2	0.89 (p NS)	21.6 vs 17.8	0.89 (p NS)
			Folfox4/Cx	69	11.9 vs 5.7	0.22 (*p* < 0.001)	26 vs 15.7	0.43 (*p* < 0.006)
Modest 2013 [65]	AIO KRK 0104 (phase II)	**6wk, ≥20%**	CT/Cx	59	8.9 vs 4.7	0.37 (*p* < 0.001)	31.6 vs 15.8	0.48 (*p* = 0.005)
Giessen 2013 [75]	FIRE-1 (phase III)	**7wk, ≥20%**	FU/irinotecan/oxaliplatin	46.8	9.9 vs 6.1	0.78 (*p* = 0.029)	27.5 vs 17.8	0.58 (*p* = 0.002)
Suzuki 2012 [76]	NORDIC VI (phase III)	**8wk, ≥ 10%**	FLIRI	57	NR	HR NR (*p* < 0.001)	NR	HR NR (*p* < 0.001)
			Lv5FU2-IRI	63				
Cremolini 2015 [70]	TRIBE (phase III)	**8wk, ≥ 20%**	Folfiri/Bv	51	12.7 vs 10	0.66 (*p* < 0.001)	35.8 vs 22.4	0.54 (*p* < 0.001)
			Folfoxiri/Bv	64				

∼ :about HRs: adjusted HR NB:not reported NS: not significant ND: not defined mo: months CT: chemotherapy Cx: cetuximab Bv : bevacizumab Folfiri: fluorouracil+leucovorin+irinotecan Folfox4: fluorouracil+leucovorin+oxaliplatin Lv5FU2-IRI: fluorouracil+leucovorin+irinotecan (de Gramont schedule) FLIRI: fluorouracil (bolus)+leucovorin+irinotecan

Although a number of studies suggest that ETS has a potential value in CRC patients, the evaluation of the prognostic role of ETS may be affected by at least two methodological problems. Firstly, it is very difficult to disentangle the role of ETS from the role of response *per se*: if early responders are compared with all remaining patients, the latter will include both late responders and patients who never respond. Therefore, the comparison should be limited to patients who eventually respond, matching early complete responders to late complete responders, and early partial responders to those who experience a partial response later. However, it is possible that some patients that partially respond early may become complete responders at subsequent examinations, further complicating the interpretation of the results. Secondly, only comparisons strictly based on landmark analyses are valid. In such analyses only the events occurring after the last response assessment are considered.

### New response parameters: extent

When using RECIST criteria, a tumor shrinkage that exceeds 30% is defined as PR. In such scenario, both a 35% overall reduction in tumor size and a 95% decrease of cancer burden would equally account for a response; it is quite evident that RECIST criteria are not capable of capturing the extent of tumor shrinkage, that might be associated with a different prognosis. Deepness of response (DoR) is another very interesting new measure of activity used to explain the impact of different levels of tumor shrinkage on survival. DoR, also called depth of response, is defined as the percentage of tumor shrinkage, in terms of longest diameter (LD) or calculated tumor volume, observed at the nadir compared to baseline [77]. The deepest response point may occur anytime before disease progression and does not need to be confirmed. DoR may be intended as a continuous variable since the tumor shrinkage may range from 0 to 100%. Alternatively, DoR may be considered as an ordinal variable, usually with five levels based on quintile distribution. A comparison between DoR, ETS and objective response characteristics is presented in Table 3.

**Table 3:** Comparison between Objective Response, Early Tumor Shrinkage and Deepness of response characteristics

	OR Objective Response	ETS Early Tumor Shrinkage	DoR Deepness of response
**Significance**	The proportion of patients with tumor size reduction of a predefined amount and for a minimum period of time	The proportion of patients who experienced a predefined relative decrease of tumor size at the very first radiological assessment	The percentage of tumor shrinkage, in terms of longest diameter (LD) or calculated tumor volume, observed at the nadir compared to baseline
**Time of assessment**	Anytime	At a prespecified early point time(6–8 weeks since treatment start)	Anytime
**Confirmation**	Needed	Not Needed	Not Needed
**Variable evaluation**	Binary or in 4 classes	Continuous or in binary	Continuous or in 5 classes

**Figure 1:**
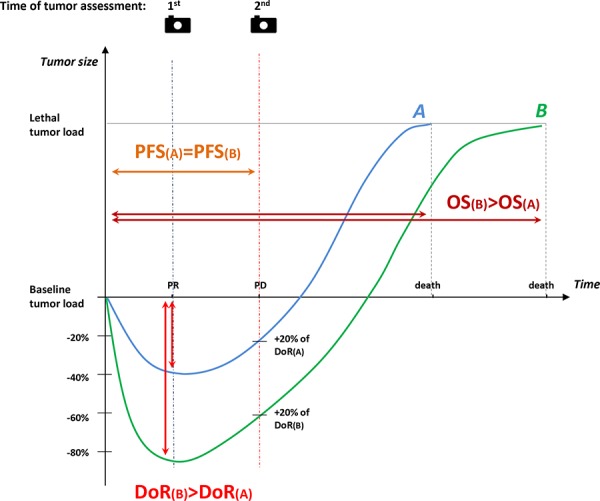
Role of Deepness of Response in the relationship between Response Rate, Progression-free Survival, and Overall Survival: a representation of the theoretical model proposed by Mansmann At 1st tumor assessment (blue dashed line), for example after 8 weeks, partial response is recorded for both patient A (−40%) and patient B (−85%). At 2nd tumor assessment (red dashed line) progression of disease is documented for both. Thus, the two patients have the same PFS (PFS(A) = PFS(B), orange solid line). Nevertheless, the time required to achieve lethal tumor burden in patient B who experienced a deeper response is longer than in patient A (OS(B) > PFS(A)). 1st = first tumor assessment; 2nd = second tumor assessment; blue curve = patient A; green curve = patient B; DoR(A) = Deepness of Response of patient A; DoR(B) = Deepness of Response of patient B; OS(A) = Overall Survival of patient A; OS(B) = Overall Survival of patient B; PD = Progressive Disease; PFS(A) = Progression-Free Survival of patient A; PFS(B) = Progression-Free Survival of patient B; PR= partial response.

From a clinical point of view, even if a maximal tumor shrinkage is not the primary treatment purpose in all cases of mCRC patients, a complete disease removal after downsizing by chemotherapy may give the potential of long-term survival or cure in potentially resectable metastatic patients. Similarly, symptomatic patients with aggressive or extensive disease may benefit from a very active first-line treatment with a high likelihood to induce disease regression in short time and produce symptoms’ relief in patients presenting with greater tumor burden. Conversely, older patients or those with major comorbidities or with limited risk for rapid deterioration may be considered for a less intense and forceful treatment strategy. In advanced colorectal cancer setting, “the deeper the response, the better the outcome” paradigm, although intuitively acceptable, has not be fully substantiated. In hematological diseases, a serum disease reduction of > 90% is defined as a very good partial response (VGPR). Extensive evidence supports the relationship between achievements of VGPR after transplant with substantially prolonged survival in previously untreated myeloma patients. This depth of response threshold not only has a clear prognostic value [78, 79] but it also may be used as a treatment guide [80]. The role of DoR as a predictor of efficacy was first analyzed by revising radiologic data of patients enrolled in CRYSTAL and OPUS trials. Results supported a prognostic relationship between increased DoR and longer survival post progression (PPS) in 841 patients with KRAS wild-type metastatic CRC treated with oxaliplatin or irinotecan-based chemotherapy regimens with or without cetuximab (* p* < 0.0001 for CRYSTAL and *p* < 0.005 for OPUS). In both trials, the median DoR was higher in patients treated with regimens containing cetuximab than in those treated with chemotherapy alone (50.9% versus 33.3%, *p* < 0.0001 in CRYSTAL; 57.9% versus 30.7%, *p* = 0.0008 in OPUS) [77]. Data about DoR were recently reported also from the PEAK study. Greater median DoR was observed with mFOLFOX6 plus panitumumab compared to mFOLFOX6 plus bevacizumab (65% [interquartile range {IQR}: 48–87%] vs 46% [IQR: 29–62%]; *p* = 0.0007) [74]. In the TRIBE study, 484 out of 508 randomized patients were evaluated to confirm whether DoR correlates with PFS and OS. Although DoR was analyzed as a continuous variable, the cut-off level used to determine a DoR was 38.9% and corresponded to the median value of the tumor shrinkage. DoR greater than 38.9% was more frequently observed in patients assigned to the FOLFOXIRI plus bevacizumab arm (58% versus 42%, *p* = 0.008). Moreover, a DoR greater than the median value was associated with longer PFS (13.1 months versus 9.3 months, HR 0.61, 95%CI 0.49–0.73, *p* < 0.0001] and OS [36.8 months versus 21.3 months; HR 0.47, 95%CI 0.35–0.58, *p* < 0.0001] [69]. These findings support the hypothesis that more profound tumor shrinkage may have a positive impact on disease outcome and may be a clinically relevant objective independently from the conversion intent, delaying tumor progression and eventually translating into significant survival advantage. Recently, the importance of DoR has been advocated to explain the results of FIRE-3 trial. In this important phase III study, although RR and PFS were similar in the two arms a significant survival increase was unexpectedly reported in the group of patients assigned to the cetuximab-containing arm [81]. A possible explanation for this difference is provided by the Authors of the trial themselves who propose a theoretical model in which patients in the cetuximab arm have DoR greater than the median value, thus requiring a longer period of time to reach a lethal tumor burden (Figure 1). At present, the possible relationship between DoR and tumor burden still remains one of the major unsolved issue and this model seems, at least in part, simplistic and questionable. Tumors are a dynamic process composed of a heterogeneous population of cells that are under continuous drug selection pressure [82, 83]. In addition, this model assumes that the tumor burden curves evolve similarly after tumor progression in both treatment arms and does not explain why median PFS values are not equally modified. It is difficult to believe that factors such as the impact of post-progression treatment, the development of new lesions and patient's existing or new comorbidities do not influence post-progression survival [84]. DoR evaluation inevitably depends on the time of tumor assessment, and the exact nadir in terms of shrinkage is unknown. Waterfall plots are vertical histograms in which bars correspond to some degree of tumor growth or shrinkage [85]. Waterfall plots are frequently used as graphical illustrations to display the magnitude of each individual patient's response to a particular drug based on a parameter, such as tumor burden. Although these graphic models have rapidly garnered optimism because they may simply and intuitively represent results for individual patients, they are also subject to substantial variability, may be influenced by measurement errors, and should be generated by experienced radiologist before being interpreted in the context of clinical trials [86].

## CONCLUSIONS

In this narrative review we have reported recent data on the value of early tumor shrinkage and deepness of response, and we have discussed how these response measures have recently emerged as novel potential surrogates in advanced stages of colorectal cancer.

Generally, patients with ETS achieve a rapid decrease in tumor size at the very first radiological assessment since commencing the treatment. DoR, instead, is a measure of the width of the response, which would enable to separate among patients achieving response those with substantial tumor shrinkage from those with smaller tumor burden decrease. Similarly to objective response, ETS and DoR have multiple potential applications. These novel response parameters may indicate drug activity, may be considered surrogate endpoints for survival in clinical trials, may trace individual benefit in a single patient or may serve as treatment guidance. ETS is evaluated either as continuous or binary (≥20% vs <20%) variable at a specific time-point. Similarly, DoR is considered either as continuous or ordinal variable, with five levels based on quintile distribution. Retrospective radiological imaging review of patients enrolled in phase III trials has suggested that these parameters may correlate with improved clinical outcomes. Although compelling, the actual role of ETS and DoR is yet to be confirmed; moreover, since objective response, ETS and DoR frequently include the same patients, it remains uncertain whether their use might increase the objective response surrogacy. Besides, their surrogacy with overall survival should be further investigated within trials with larger sample size and appropriate statistical analyses. Interestingly, data from the TRIBE study suggested that an early and more profound tumor shrinkage to FOLFOXIRI and bevacizumab might consistently correlate with both increased PFS and prolonged OS, thus providing a springboard for future reasoning. Although the evaluation of non-cytotoxic drugs may complicate the scenario, these new parameters of response deserve to be further studied as surrogate endpoint in clinical trials, interim analyses of phase III studies and trials with adaptive design.
